# Medication Administration Error Reporting and Associated Factors among Nurses Working in Public Hospitals, Ethiopia: A Cross-Sectional Study

**DOI:** 10.1155/2021/1384168

**Published:** 2021-05-05

**Authors:** Kokebie kefelegn Asefa, Deguale Dagne, Wassie Negash Mekonnen

**Affiliations:** ^1^DebreBerhan University College of Health Science, Department of Nursing, Debre Berhan, Ethiopia; ^2^DebreBerhan University College of Health Science, Department of Public Health, Debre Berhan, Ethiopia

## Abstract

**Background:**

Medication administration error is one of the most common errors that occur when a discrepancy occurs between the drugs received by the patient and the drug intended by the prescriber. A lot of studies were conducted on medication administration error. But there were a few studies on whether those medication administration errors are reported or not among nurses in Ethiopia. So this study is aimed at assessing the magnitude of medication administration error reporting and the associated factors among nurses.

**Objectives:**

To assess the magnitude of reported medication administration error and associated factors among nurses working in public hospitals, Ethiopia.

**Methods:**

An institutional-based cross-sectional study design was employed from March to April 2019. Simple random sampling technique was used. A structured self-administered questionnaire was used to collect the data. Data were entered using EpiData version 3.1 and descriptive analysis, bivariate, and multivariate logistic regression analyses were carried out using SPSS version 21 software.

**Results:**

The magnitude of medication administration error reporting was found to be 37.9%. Being female [adjusted odds ratio (AOR) = 2.91; confidence interval (CI) (1.45–5.85)]; belief that errors should not be reported [AOR = .3; CI (.15–.61)]; having work experience of greater than 15 years [AOR = 3.4; CI (1.11–13.85)]; having bachelor science degree [AOR = 3.27; CI (1.61–6.66)]; and caring for greater than 10 patients [(AOR = .4; CI (.16–.96)] were factors associated with nurses medication administration error reporting.

**Conclusion:**

The magnitude of medication administration error reporting among nurses was found to be low. To increase medication administration error reporting, efforts should be made to change the attitude of nurses on the belief that errors should be reported, retaining staffs that have longer experience, upgrading staffs educational status, and limiting the number of patients cared by a single nurse.

## 1. Background

According to World health organization (WHO) 2017 report, globally the cost associated with medication errors has been estimated about 42 billion US dollars annually [1]. The United States National Coordinating Council for medication error reporting and prevention defines a medication error as “any preventable event that may cause or lead to inappropriate medication use or patient harm while the medication is in the control of the health care professional, patients, or consumer.” Such events may be related to professional practice, health care products, procedures, and systems including prescribing, order communication, product labeling, packaging, nomenclature, compounding, dispensing, distribution, administration, education, monitoring, and use [2].

Medication administration error (MAE) is one of the most common errors in the medication error process and occurs when a discrepancy occurs between the drugs received by the patient and the drugs intended by the prescriber [3]. To improve patient safety and reduce the incidence of MAE, nurses should intercept medication errors before reaching the patient by adhering to the six rights of medication administration. The six rights of medication administration are the right patient, right drug, right time, right route, right dose, and right documentation. Reporting of MAEs is fundamental to error prevention. Reporting reduces the adverse effects of errors and effectively helps to avoid future errors that can cause patient harm. In addition, reporting of MAEs reduces the number of future errors, diminish personal suffering, and decrease financial costs [4].

Voluntary reporting is a critical strategy to detect MAEs. A critical strategy to reduce MAEs is to use error detection, which comprises error recognition and reporting. MAE reporting requires professionals to recognize the occurrence of MAEs and to report them through official channels. MAE reporting is an effective way used to identify the root causes of MAE and to prevent repeating them in future [5]. When MAEs occur, their effects can be mitigated by facilitating correct actions, use of antidotes, and use of appropriate guidelines. Additionally, further education and training will be provided to improve work competencies [6].

A study conducted in North Carolina, Philippines, and Saudi Arabia showed that about 37.9%, 52%, and 28.6% of study participants were reporting MAE, respectively [7–9].

A study conducted in Ethiopia indicated that the proportion of MAE reporting among nurses was found to be 57.4% [10]. Another study conducted in University of Gondar Referral Hospital, Ethiopia, revealed that the estimated MAE reporting was found to be 29.1%. The perceived rates of MAEs reporting for non-intravenous-related medications ranged from 16.8% to 28.6% and for intravenous-related medications from 20.6% to 33.4% [11].

There are a lot of studies done on MAE [12–17], but to the knowledge of the researcher, there are only a few studies [10, 11] reporting whether those MAEs are reported or not among nurses in Ethiopia. In developing countries like Ethiopia, educational, economic, and trained manpower problems, the issue is primarily one of the least investigated and neglected health problems [18]. So, this study is aimed at answering the research questions, what is the magnitude of medication administration error reporting among nurses? And what factors are associated with nurses' medication administration error reporting?

## 2. Methods

### 2.1. Study Area, Period, and Design

The study was conducted in public hospitals of North Shoa Zone, Amhara region, Ethiopia. North Shoa is one of the 10 zones in Amhara region. In North Shoa Zone, there are 9 public hospitals. This study was conducted on three hospitals from March to April. An institutional-based cross-sectional study design was employed.

### 2.2. Source Population and Study Population

All nurses working in public hospitals of North Shoa Zone were used as a source population. Nurses that work in selected public hospitals of North Shoa Zone were used as a study population.

### 2.3. Inclusion and Exclusion Criteria

All nurses who have a minimum of diploma qualification in nursing and involved in direct patient care, those who have at least six months of work experience, and those who are full time workers were included. Those nurses who were on annual leave, maternal leave, seriously ill, and attending external training courses off-site at the time of the data collection were excluded.

### 2.4. Sample Size Determination and Sampling Procedure

The sample size was determined using single population proportion formula with the assumption of 95% confidence interval with margin of error of 5%, 10% non-response rate and 57.4% of prevalence of medication administration error reporting from a study conducted in Addis Ababa [10]; the sample size becomes 376. Since the source population is 472 which is less than 10,000, using finite population correction formula and adding 10% non-response rate, the final sample was 230.

To select 230 nurses from the total of nine hospitals, three hospitals were selected by using simple random sampling. Then, the sample size was proportionally allocated to the number of nurses in each hospital. Finally, study participants were selected by using simple random sampling technique.

### 2.5. Data Collection Method and Procedures

The instrument used for data collection was a structured self-administered questionnaire. The questionnaire was adapted and modified from a previous study [10]. It contains 48 questions arranged into six sections.

The first section deals with the sociodemographic characteristics of the participants; the second section contains work-related aspects of nurses; the third section is regarding knowledge on MAEs; the fourth section is about the reason why MAEs occur; the fifth section is the reason why MAEs are not reported; and the six section deals with the percentage of each type of error reported. The questionnaire that is used in this study is available as a supplementary file.

To assess the validity of the instrument, face validity and content validity were done by five experts. Content validity ratio (CVR) and content validity index (CVI) were measured and were 0.2 and 0.83, respectively, which shows that the instrument is valid. The reliability of the instrument was checked using Cronbach's alpha and was 0.8 which showed that the instrument was reliable. The questionnaire was pretested on 5% of the sample size at the nearby hospital and appropriate amendment was done on it.

Data were collected by three diploma holder nurses. Training was provided for data collectors about the overall objective of the study, content of the questionnaire, and how to collect the data. The questionnaire was given to the randomly selected participants. Confidentiality of the information was kept by excluding the names of the respondents and names of the hospitals in the questionnaire.

### 2.6. Data Analysis

The returned questionnaires were checked for completeness, cleaned and entered into EpiData 3.1, and analyzed using SPSS (Statistical Package for the Social Sciences) version 21. Descriptive analysis was done and presented using tables and texts. Bivariate and multivariate logistic regression analysis was used to identify factors associated with medication administration error reporting. Variables with *p* < 0.02 in bivariate logistic regression analysis were entered to multivariate logistic regression analysis [13]. The adjusted odds ratio was used to interpret the strength of association at 95% CI and those variables with *p* < 0.05 in multivariate logistic regression analysis were considered as significant predictors of the outcome variable.

## 3. Results

### 3.1. Sociodemographic Characteristics

This section gives an overview of the sociodemographic characteristics of nurses working in public hospitals of North Shoa Zone, Amhara, Ethiopia, 2019. From the survey, information about sex, age, marital status, educational level, and educational degree attained of the respondents was analyzed.

Out of 230 proposed study participants, 224 nurses participated in this study indicating a response rate of 97.4%. More than half (120) (53.5%) of respondents were females, 117 (52.2%) of them were married, 104 (46.4%) of nurses were in the age group of 25–29 years old, 120 (53.5%) of nurses had Bachelor of Science in Nursing and received their degree from a governmental institution, 195 (87.1%) (See Table 1).

### 3.2. Work-Related Characteristics of the Respondents

From the participants, 106 (47.3%) had a work experience of ≤4 years, 157 (70.1%) nurses worked in the inpatient department, 143 (63.8%) nurses worked in the day duty shift, and 119 (53.1%) of them worked for 3–6 months on their unit. Regarding average patient care, 81 (36.2%) of the participants provided care for 1–6 patients. The majority of the participants or 189 (84.4%) nurses responded that there is no MAE reporting system in their hospital (see Table 2).

### 3.3. Magnitude of Medication Administration Error Reporting among Nurses

The proportion of MAE reporting in the last six months that was committed or witnessed among nurses in this study was found to be 85 (37.9%). Out of the reported MAEs (*n* = 85), about 59 (69.4%) of medication administration error reporting was found among female nurses as compared to male ones (26) (30.6%).

More than half (132) (58.9%) of the participants perceived that MAEs should be reported as they occur. Out of the total participants (*n* = 224), majority (205) (91.8%) of the study participants communicate with other nurses when they faced doubt during medication administration and 210 (93.8%) of the participants believed that the 6 rights in medication administration would avoid errors in medication administration (see Table 3).

Among the reasons for MAEs, about 122 (54.5%) of the respondents said that physician orders were not clear/legible, 133 (59.4%) said change of physician orders frequently, 127 (56.7%) said failure of pharmacists to label the medication correctly, 133 (59.4%) of them said the situation in which many patients are on the same or similar medications, 130 (58.0%) of them said the situation in which unit staff do not receive enough service training on new medications, and 136 (60.7%) of them responded that inadequate unit staffing was identified as a reason for MAE.

Regarding the reasons why medication administration errors were not reported, 142 (63.4%) of respondents expressed their disagreement with hospital's definition of a medication error, 121 (54.0%) sampled nurses did not think the error is important enough to be reported, 143 (63.8%) participants believed the expectation that medications should be given exactly as ordered is unrealistic. Another reason for not reporting MAEs is that about 132 (58.9%) nurses have fear of adverse consequences from reporting medication errors and 145 (64.7%) of respondents believed nursing administration focuses on the individual rather than looking at the system as a potential cause of the error.

### 3.4. Percentage of Each Type of Error Reported

The types of medication administration errors reported among nurses were measured by the frequency of wrong route, wrong time, wrong patient, wrong dose, wrong drug, and medication is omitted; out of the sampled 224 nurses, 155 (69.2%) of them responded wrong route of administration, 126 (56.3%) of the respondents opt wrong time of administration, 167 (74.6%) of respondents responded wrong patient administration, 99 (44.2%) respondents provided wrong dose, 162 (72.3%) of them administered wrong drug, and 121 (54.0%) respondents were not given prescribed medications (see Table 4).

### 3.5. Factors Associated with Nurses' Medication Administration Error Reporting

Binary logistic regression analysis was done to identify factors associated with nurses' MAE reporting. Sex, educational status, educational award, nurses work experiences, average patients care per shift, belief that errors should be reported, the names of many medications being similar/look alike, not agreeing with hospital's definition of a medication error, and fear of adverse consequences from reporting medication errors had an association with MAE reporting in bivariate logistic regression analysis. All variables that have an association with the outcome variable at *p* < 0.2 in bivariate logistic regression analysis were included in the multivariate logistic regression analysis model. In multivariable logistic regression analysis, factors that were significantly associated with nurses' MAE reporting were sex, educational status, working experience, belief that errors should be reported, and average patient care.

The proportion of MAE reporting was higher among female nurses as compared to male ones. Female nurses were almost three times more likely to report MAEs than male nurses [AOR = 2.91; CI (1.45–5.85)]. Similarly, educational status was an important predictor of MAE reporting. BSc nurses were more than three times more likely to report medication administration errors as compared to those who are diploma nurses [AOR = 3.27; CI (1.61–6.66)]. And MSc nurses were more than six times more likely to report MAEs than Diploma nurses [AOR = 6.4; CI (1.02–40.3)].

Regarding with working experience, participants who worked greater than 15 years were almost four times [ AOR = 3.93; CI (1.11–13.85)] more likely to report MAEs than those who work less than than or equal to four years. Participants who gave care for greater than 10 patients were 0.4 times less likely or 60% times more likely to report than those participants who gave care for less than or equal to 6 patients (AOR = .4; CI (0.16–.96)). Participants who believed an error should not be reported were 0.3 times less likely or 70% times [AOR = .3; CI (.15–.61)] more likely to report MAEs than those participants who believed that errors should be reported (see Table 5).

## 4. Discussion

This study was carried out with the aim of determining the magnitude of MAE reporting and the associated factors. In this study, the proportion of MAE reporting was low. This was in line with the finding in North Carolina which indicated that 37.9% of the participants reported all types of medication errors that occurred on their unit [7] and in a study in Canada, 42.9% (*n* = 506) have reported a near miss to the resident safety program, 45.7% (*n* = 539) have reported a minor error, 21.3% (*n* = 141) have reported a serious error, and 11.9% (*n* = 141) have never reported an error [19]. However, the finding of this study was lower than a study done in Addis Ababa federal ministry level hospitals, Ethiopia (57.4%) [20]. This implies that the habit of reporting MAEs is low. Hence, that all types of errors should be reported. This may be due to lack of readily available reporting system among the hospitals under the study. Additionally, there is also variation in the type of hospitals for the study in which the study done in Addis Ababa federal ministry level hospitals was conducted in three specialized hospitals, whereas this study was done in one referral hospital and two primary hospitals [10].

The finding of this study was slightly high as compared to studies in Saudi Arabia and University of Gondar Referral Hospital in which 28.6% and 29.1% of MAEs were reported, respectively [11, 20], and higher than a study in tertiary hospitals in Addis Ababa in which 13.4% of MAEs were reported [10]. The possible reason for the difference may be due to the differences in organizational medication administration error reporting systems and differences in the time frame that the studies were conducted. Additionally, they may fear legal issues, blame for the reported errors in the working environment, and fear lack of job following the reporting of errors [10].

In this study, the proportion of female nurses who reported medication errors was higher than the male nurses and was statistically significant. Female nurses were almost three times more likely to report MAEs than male nurses. The result was consistent with that of a study from Addis Ababa [10]. This difference may be due to the fact that in this study female nurses face more interruption 77 (64.7%) than male nurses. So, they may make more errors and report more.

Educational status was an important predictor of MAE reporting. BSc nurses are more than three times more likely to report MAEs as compared to those who are Diploma nurses. MSc nurses were more than six times more likely to report than Diploma nurses. The result was consistent with that of the study from Addis Ababa, Gondar [7, 10, 11] in which participants who had educational status of BSc and above were more than one times more likely reported MAE than those participants who had educational status of diploma. It is also in line with a study in Canada in which having a higher level of education is an independent predictor of disclosing more information about the errors. This is possibly due to the fact that those participants who had higher educational status may have higher knowledge, attitude, and practice toward the drug adverse effect, and know more about the code of ethics through their educational journey.

From the participants, 58.9% perceived that errors should be reported as they occur for the safety of patients and this is lower than the study from Addis Ababa. The possible difference may be due to lack of a readily available practice system of MAE reporting [10].

Participants who say medication administration errors should not be reported were 70.1% times less likely to report MAEs than those who say medication administration errors should be reported.

This result is lower than the previous study conducted in Addis Ababa [10].

Pertinent to work experience, participants who worked greater than fifteen years were almost four times more likely to report medication administration errors than those who worked less than or equal to four years. This result is consistent with the study conducted in Saudi Arabia [20]. This is possibly due to the fact that nurses who work longer may be concerned about the improvement of quality of service rather than the consequence of reporting medication administration errors on their career but if the nurses are new and have a short period of experience, they may be concerned about loss of their career and fear blame of their errors.

Participants who gave care for greater than 10 patients were 60.4% times less likely to report medication administration errors than those who gave care for less than or equal to 6 patients. This result is contradicted with a study conducted in Saudi Arabia [20]. This might be due to difference in time frame in which the study was conducted and difference in organizational (hospital) type.

The result of this study shows that medication administration error reporting among nurses was low. This implies that there is a problem in nursing practice. So, each hospital should create and apply a reporting system and nurses should practice the documentation and reporting of errors through the reporting system.

As a limitation, since the study was done by cross-sectional study design, it does not determine cause effect relationship. The number of the participants might have contributed to the absence of a significant association between some of the factors and MAE reporting, as well as to the generalizability of the findings.

## 5. Conclusion

The magnitude of MAER among nurses was found to be low. Being female, belief that errors should be reported, working experience, educational status, and average patient care were factors significantly associated with nurses' medication administration error reporting. To increase medication administration, error reporting efforts should be made to change the attitude of nurses on the belief that errors should be reported, retaining staffs that have longer experience, upgrading staff educational status, and limiting the number of patients cared by a single nurse.

## Figures and Tables

**Table 1:** Socio-demographic characteristics of nurses working in public hospitals of North 53 Shoa Zone, Amhara, Ethiopia, 2019 (*n* = 224).

Variables	Responses	Frequency (*n* = 224)	Percentage (100%)
Sex	Male	104	46.5
	Female	120	53.5

Age	20–24	15	6.7
	25–29	104	46.4
	30–34	48	21.4
	≥35	57	25.5

Marital status	Single	96	42.9
	Married	117	52.2
	Others	11	4.9

Educational status	Diploma	93	41.5
	BSc	120	53.5
	MSc	11	5.0

Educational degree attained	Government institution	195	87.1
	Private institution	29	12.9

**Table 2:** Work-related characteristics of nurses in North Shoa Zone public hospitals 56 Amhara, Ethiopia, 2019.

Variables	Response	Frequency	Percentage
Working experience	≤4 years	106	47.3
	5–10 years	71	31.7
	11–14 years	27	12.1
	≥15 years	20	8.9

Working unit	Medical ward	59	26.3
	Surgical ward	50	22.3
	Pediatrics ward	23	10.3
	Obstetrics and gynecology ward	9	4.0
	Emergency	28	12.5
	Intensive care unit	16	7.1
	Outpatient department	18	8.0
	Others	21	9.4

Duration on present unit	≤3 months	15	6.7
	3–6 months	119	53.1
	≥6 months	90	40.2

Current duty shift	Day shift	143	63.8
	Night shift	61	27.2
	Alternative shift	20	8.9

Average patient care	1–6 patients	81	36.2
	7–10 patients	76	33.9
	>10 patients	67	29.9

Presence of MAE reporting system	Yes	35	15.6
	No	189	84.4

Others = NICU, OR, TB.

**Table 3:** Magnitude of MAE reporting among nurses working in public hospitals of North Shoa Zone, Amhara, Ethiopia, 2019 (*n* = 224).

Variables	Response	Frequency	Percentage
Report MAE	Yes	85	37.9
	No	139	62.1

Should medication errors be reported	Yes	132	58.9
	No	92	41.1

Communicate with another nurse when facing doubt during medication administration	Yes	201	89.7
	No	23	10.3

Following 6 rights of medication administration avoids MAE	Yes	205	91.5
	No	19	8.5

**Table 4:** Types of medication administration errors reported among nurses working in public hospitals of North Shoa Zone, Amhara, Ethiopia, 2019 (*n* = 224).

Variables	Value	Frequency	Percentage
Wrong route	1–20	155	69.2
	21–30	43	19.2
	31–40	16	7.1
	41–50	7	3.1
	>50	3	1.3

Wrong time	1–20	126	56.3
	21–30	55	24.6
	31–40	21	9.4
	41–50	12	5.4
	>50	10	4.5

Wrong patient	1–20	167	74.6
	21–30	32	14.3
	31–40	14	6.3
	41–50	7	3.1
	>50	4	1.8

Wrong dose	1–20	99	44.2
	21–30	73	32.6
	31–40	25	11.2
	41–50	17	7.6
	>50	10	4.5

Wrong drug	1–20	162	72.3
	21–30	36	16.1
	31–40	15	6.7
	41–50	8	3.6
	>50	3	1.3

Medication is omitted	1–20	121	54.0
	21–30	52	23.2
	31–40	23	10.3
	41–50	15	6.7
	>50	13	5.8

**Table 5:** Bivariate and multivariable logistic regression analysis of factors associated with 278 nurses MAE reporting working in public hospitals of North Shoa zone, Amhara, Ethiopia, 2019.

Variables	Response	Medication administration error reporting		Odds ratio (95% CI)	
		Yes	No	Cor	AOR
Sex	Male	26 (24.8%)	79 (75.2%)	1.00	1.00
	Female	59 (49.6%)	60 (50.4%)	2.98 (1.68–5.28)	2.91 (1.45–5.85)^*∗*^

Educational status	Diploma nurse	21 (21.9%)	75 (78.1%)	1,00	1.00
	BSc nurse	56 (47.5%)	62 (52.5%)	3.22 (1.76–5.90)	3.27 (1.61–6.66)^*∗*^
	MSc nurse	8 (80.0%)	2 (20.0%)	14.28 (2.81–72.42)	6.40 (1.02–40.30)^*∗*^

Working experience	≤4 yrs	38 (35.8%)	68 (64.2%)	1.00	1.00
	5–10 yrs	28 (39.4%)	43 (60.6%)	1.35 (0.64–2.81)	1.17 (0.51–2.70)
	11–15 yrs	11 (40.7%)	16 (59.3%)	5.17 (2.40–11.13)	2.11 (0.84–5.30)
	>15 yrs	8 (40.0%)	12 (60.0%)	9.78 (3.47–27.54)	3.93 (1.11–13.85)^*∗*^

Educational award	Gov't institution	81 (41.5%)	114 (58.5%)	1.00	1.00
	Private institution	4 (13.8%)	25 (86.2%)	0.22 (0.07–.67)	0.38 (0.10–1.40)

Average patient care	1–5	41 (50.6%)	40 (49.4%)	1.00	1.00
	6–10	28 (36.8%)	48 (63.2%)	.56(,30–1.07)	0.66 (0.30–1.48)
	≥11	16 (23.9%)	51 (76.1%)	.30(.15–.62)	0.40 (0.16–.96)^*∗*^

Errors should be reported	Yes	64 (48.5%)	68 (51.5%)	1.00	1.00
	No	21 (22.8%)	71 (77.2%)	0.31 (0.17–.57)	0.30 (0.15–.61)^*∗*^

Agree with hospital definition on MAEs	Agree	29 (59.2%)	20 (40.8%)	1.00	1.00
	Disagree	56 (32.0%)	119 (68.0%)	3.08 (1.60–5.90)	1.40 (0.60–3.23)

Fear adverse	Agree	58 (44.6%)	72 (55.4%)	1.00	1.00
Consequence from MAE reporting	Disagree	27 (28.7%)	67 (71.3%)	0.50 (0.28–.88)	0.97 (0.47–1.99)

NB: variables having a *p* value ≤ 0.2 in bivariate analysis included in the multivariable analysis. *∗*Statistically significant at *p* value < 0.05.

## Data Availability

All the data are available from the corresponding author on reasonable request.

## Abbreviations

DBRH: Debre Berhan Referral Hospital
DBU: Debre Berhan University
ENA: Ethiopia Nursing Association
FDA: Food and Drug Agency
ICU: Intensive care unit
IV: Intravenous
MAEs: Medication administration errors
MAER: Medication administration error reporting
MOH: Ministry of Health
MSc: Master of Science
NICU: Neonatal intensive care unit
OPD: Outpatient department
OR: Operation room
SPSS: Statistical Package of Social Sciences
TB: Tuberculosis
UGRH: University of Gondar Referral Hospital
US: United States
WHO: World Health Organization.
